# Correlation of Biomechanical Variables of Lower Extremity Movement During Functional Tests and Tasks in Youth League Football Players: Cross-Sectional Correlation Study

**DOI:** 10.2196/69046

**Published:** 2025-07-10

**Authors:** Anna Davidoviča, Sergejs Davidovičs, Guna Semjonova, Alexei Katashev, Alexander Oks, Linda Lancere, Signe Tomsone, Maksims Zolovs

**Affiliations:** 1 Department of Rehabilitation, Riga Stradiņš University, Anniņmuižas bulvāris 26A, Riga, Latvia, 371 25637767; 2 Institute of Mechanical and Biomedical Engineering, Riga Technical University, Riga, Latvia; 3 Institute of Architecture and Design, Riga Technical University, Riga, Latvia; 4 Department of Sociotechnical Systems Modelling, Vidzeme University of Applied Sciences, Valmiera, Latvia; 5 Statistics Unit, Riga Stradiņš University, Riga, Latvia; 6 Institute of Life Sciences and Technology, Daugavpils University, Daugavpils, Latvia

**Keywords:** biomechanics, youth athletes, wearable technology, postural control, muscle activity, center of pressure, sports injury prevention, wireless sensors, functional movement

## Abstract

**Background:**

Football is the most widely played sport globally but carries a high risk of lower limb injuries, particularly among youth athletes. Real-time biomechanical monitoring can play a critical role in injury prevention. However, traditional lab-based systems are often complex and impractical for field use. Recent advances in wearable technology, such as inertial sensors and smart socks, provide more accessible solutions for movement analysis. The DAid smart sock system is a promising tool, but further evidence is needed to support its use in lower extremity functional assessments.

**Objective:**

This correlational study aimed to investigate the correlation between lower limb joint angles, muscle activity, and plantar pressure distribution during the “Single Leg Squat” (SLS) and its variations in youth football players, using wireless wearable sensors in a field-based setting.

**Methods:**

In total, 32 youth football players (16 male and 16 female individuals; mean age 14.6, SD 0.5 years) performed SLS movements while wearing the NOTCH inertial motion sensors, DAid smart socks (plantar pressure), and PLUX muscleBAN EMG system. Spearman correlation was used to explore relationships between hip, knee, and ankle joint kinematics; muscle activity (gluteus medius, gluteus maximus [GMx], vastus lateralis, and biceps femoris); and changes in center of pressure (COP) on the plantar surface.

**Results:**

A strong positive correlation was found between hip adduction and medial foot pressure (COP1X: ρ=0.785, *P*<.001). Knee flexion was strongly correlated with gluteus medius (ρ=0.809) and GMx (ρ=0.841) muscle activity. Hip internal rotation also showed moderate to strong correlations with both COP variables (COP1Y: ρ=0.585) and GMx activation (ρ=0.477). Significant gender-specific differences were identified: male individuals showed stronger correlations between joint angles and muscle activation, while female individuals demonstrated a strong correlation between knee flexion and overall plantar pressure (COP2W: ρ=0.818). Several moderate correlations (0.35<ρ<0.47) further confirmed interactions between joint movement, muscle activity, and plantar pressure.

**Conclusions:**

The findings support the feasibility and utility of wireless wearable sensors—including inertial measurement units and smart socks—for in-field biomechanical analysis in youth football players. The study confirmed key relationships between joint mechanics and plantar pressure distribution, suggesting their relevance in injury risk screening. The DAid smart sock system, in particular, demonstrated reliable performance for assessing medial-lateral loading patterns associated with hip and knee movement. These insights may help guide neuromuscular training and individualized injury prevention strategies in young athletes.

## Introduction

Football is the most widely played sport globally, with around 400 million participants across 208 countries [1]. Among the 38 million registered players, over half are under 18 years old [2]. Due to its high-intensity nature, football ranks among the top 5 sports with the highest injury risk [2,3]. Injuries commonly involve the lower extremities, particularly the thighs, ankles, knees, and hips, with frequent issues including muscle damage, strains, and contusions [4]. Youth football players are especially vulnerable to injuries, particularly during growth spurts, with one in 3 players experiencing injuries each season, many of which are noncontact [3,5]. Altered movement patterns such as dynamic knee valgus are a significant biomechanical risk factor for injuries, such as anterior cruciate ligament (ACL) tears [6]. Understanding and managing this risk factor is crucial for effective injury risk reduction. Preventive exercise programs like Fédération Internationale de Football Association (FIFA) 11+ [7] have proven effective in reducing injury risk by focusing on muscle strength, balance, and dynamic stability, with studies demonstrating a 30% reduction in injury rates [1]. A key component of this program, the “Single Leg Squat” exercise, is designed to improve lower limb movement patterns and balance. However, despite the success of FIFA 11+, there remains a gap in providing personalized, real-time biofeedback on lower limb biomechanics during training, particularly concerning the functional tasks that expose youth players to injury risks [8]. Assessing an entire team simultaneously during exercises and tests limits the ability to provide personalized feedback, which is essential for progressing athletes to more advanced exercises and more intensive training sessions [8]. Coaches often provide individualized feedback during training, but this feedback can be subjective and varies based on the coach’s experience [9]. An objective approach to providing feedback can be achieved through motion-capture biofeedback systems [10-12]. While optical motion capture, Microsoft Kinect cameras [13,14], 3D kinematic analysis [15], PhaseSpace systems [16], and force plates [17] are commonly used for biomechanical assessments, they present challenges that hinder their practical application in football training. Optical motion capture systems require a fixed setup with a restricted field of view, making them unsuitable for on-field use [18,19]. Force plates, which measure ground reaction forces and foot plantar pressure, are typically confined to laboratory settings, limiting their practicality for daily football practice [20,21]. Given these limitations, wireless smart sensor systems present a promising alternative for providing real-time biofeedback on lower limb motion and biomechanics. Inertial measurement unit systems can capture data on specific body forces, angular velocity, and magnetic fields [22,23]. Wireless sensors that monitor muscle activity in real-time, using electromyographic signals, can provide insights into muscle performance and condition [24,25]. In addition, smart insole systems and smart socks, such as DAid Pressure Sock systems, measure biomechanical parameters of the foot, including the center of pressure (COP) and pressure distribution across the plantar surface [26-30]. These sensor systems offer significant benefits in sports medicine due to their cost-effectiveness, ease of use, and lightweight design, making advanced monitoring technology accessible to a broader range of sports teams and medical professionals [31,32]. However, challenges include synchronizing data from multiple sensors, signal interference, and potential restrictions on natural movement [30,33]. Previous studies have demonstrated a relationship between lower limb biomechanical variables during functional tasks. Increased knee muscle strength is associated with reduced knee valgus motion [34-38]. In addition, higher levels of lower limb muscle preactivity—particularly in the quadriceps and hamstrings—are linked to a greater peak knee valgus angle [35,39]. Regarding foot biomechanics, a positive correlation exists between greater foot pronation and increased pressure on the front to medial plantar surface during functional squat activities [21]. Considering the difficulties involved in using multiple wireless sensors simultaneously and the importance of accurately tracking foot biomechanics and limb movement, this study aims to investigate correlations between plantar pressure measurements, electrical activity of muscles, and lower extremity movement angles during functional tests like the “Single Leg Squat” in youth league football players. The hypothesis states that there will be strong correlations among key biomechanical variables—joint angles, muscular activation, and foot plantar pressure—during the “Single Leg Squat” test, using measurements from wireless sensor systems in field tests. Specifically, the position of the COP on the plantar surface is expected to be significantly associated with lower limb movement patterns and muscle activity. This study will demonstrate the relevance of wireless sensor systems for assessing lower limb biomechanics in football players, with the DAid smart sock system serving as one of the tools to facilitate this assessment.

## Methods

### Study Design

To obtain the aim of the study, the correlation research design was used, where associations between variables can be positive (variables change in the same direction), negative (variables change in opposite directions), or null (no relationship). The sample size was estimated based on Fraenkel and Wallen [40], who recommend a minimum of 30 participants for correlational studies to ensure reliable results. Given the target population and study design, a total of 32 participants was considered sufficient for meaningful statistical analysis.

### Participants

The study involved 32 youth football players (both male and female) from the Latvian Youth Football League (U-14 and U-15), recruited between September 2023 and December 2023. Participants were required to be between 14 and 15 years old, actively playing in a youth football league, and have at least 5 years of experience in football. They had to be free of pain in the hip, knee, or ankle joints during movement and voluntarily agree to participate in the study. Participants were excluded if they had any lower limb disease, deformity, injury, or surgery within the past 12 months. Those with vestibular disorders that prevented them from performing the “Single Leg Squat” test and its variations were also excluded. Additional exclusion criteria included the presence of metallic implants in areas where sensors were applied and the use of knee or ankle braces or kinesiology tape. Both limbs were evaluated in this study; however, differences between the dominant and nondominant legs were not specifically analyzed. Limb dominance was determined based on the leg preferred for kicking a ball, a commonly used method in sports biomechanics research [41]. All participants completed the “Single Leg Squat” functional test, wearing tight-fitting sportswear that allowed accurate measurements and sports shoes with appropriate insoles.

### Ethical Considerations

The study was conducted in compliance with the Declaration of Helsinki and Latvian laws, adhered to the European Union’s General Data Protection Regulation 2016/679. Before participation, the study’s procedures were explained to participants and their guardians, and informed consent was obtained. Ethical approval was granted by the Riga Stradins University Research Ethics Committee (approval number 2-PĒK-4/294/2023). Participation was voluntary, with the right to withdraw at any time, and participant confidentiality was maintained throughout the study. The participants did not receive any compensation for their participation in the study.

### Instrumentation and Data Collection

Data collection involved three main instruments: the DAid Pressure Sock System (Riga, Latvia), the NOTCH Inertial Sensor System (Notch Interfaces Inc, New York, United States), and the PLUX Wireless Biosignals system (PLUX Wireless Biosignals, Lisbon, Portugal).

### DAid Pressure Sock System

This system, equipped with 6 sensors in the sole, measured plantar pressure at up to 200 Hz per channel. The sensors were positioned under the heel, arch, and metatarsal heads to monitor gait and assess foot pressure distribution. Data were transmitted via Bluetooth to a remote device for storage. The system was calibrated using participants’ weight shifts. Activated before testing, the socks were connected via Bluetooth to “Fastreader” software in the LabView environment. The DAid records relative pressure values under each sensor to calculate the COP coordinates along the mediolateral (COPx) and anteroposterior (COPy) axes, as well as the overall COP position (COPw). More details about this system have been described in previous studies [29,30].

### NOTCH Inertial Sensor System

The NOTCH inertial measurement unit system, comprising 9-axis inertial sensors, was used to measure lower limb angles during movement. Using 6 sensors placed on the lower body, data were wirelessly transmitted to a mobile device and processed to map a 3D human skeleton. The system recorded at 40 Hz for 10-second intervals during the “Single Leg Squat” test. Sensors were calibrated and placed according to the user manual. The 6 inertial sensors were positioned at specific anatomical landmarks for accurate measurement: one on the sternum, one on the waist, and one on each thigh and calf (placed laterally at the midthigh and midcalf regions). Sensors were color-coded and placed according to the app’s guidelines. The “Lower body + hip” configuration was used, and participants followed specific positioning instructions to ensure accuracy.

### PLUX Wireless Biosignals (MuscleBAN Kit)

The PLUX Wireless Biosignals system with an electromyographic add-on was used to collect muscle electrical activity data. The system includes an electromyographic sensor, triaxial accelerometer, and magnetometer, capturing data at up to 1000 Hz with 16-bit resolution. The device, powered by an internal battery and featuring dual Bluetooth modules, provided real-time muscle activity and movement data for analysis. Four electrodes were placed to measure muscle activity in this study. Electrode placement was identified according to Surface Electromyography for the Non-Invasive Assessment of Muscles (SENIAM) guidelines [42]. Electrodes were positioned on the vastus lateralis (VL), gluteus maximus (GMx), gluteus medius (GM), and biceps femoris (BF)**.** After placing the electrodes, participants performed a maximum voluntary contraction (MVC) to evaluate muscle activity, achieved by performing maximal contractions against a stable resistance [42]. Muscle activity was recorded throughout the execution of each “Single Leg Squat” repetition, ensuring continuous data collection during movement. The recording duration per trial was 10 seconds, covering the entire squat cycle. Electromyographic signals were normalized [43], using electromyographic data recorded during MVC as the reference. The signals were processed by calculating the root mean square from the rectified signal with a 0.2-second window. The highest root mean square value from all test repetitions was used to normalize the electromyographic signals, assessing muscle activity relative to its maximum neural activation. During the “Single Leg Squat” test and its variations, the PLUX system continuously measured the electrical activity of the VL, GM, GMx, and BF muscles, providing a dynamic assessment of neuromuscular activation patterns.

### Study Procedure

Participants’ demographic and physical characteristics, such as age, height, weight, shoe size, playing position, and dominant leg, were recorded. Each participant performed the “Single Leg Squat” (SLS) and its 3 variations, completing 3 repetitions of each type (Textbox 1):

Textbox 1. **Description of Single Leg Squat (SLS) Variations: Front (SLS_F), Middle (SLS_M), and Back (SLS_B) based on non-supporting leg position while maintaining a 60° knee flexion in the supporting leg.**

- Single Leg Squat – Front: Hands placed on the hips, the non-supporting leg extended forward, and the supporting leg performing a squat to a 60-degree knee flexion angle.
- Single Leg Squat – Middle: Hands placed on the hips, the non-supporting leg positioned in the middle and bent at a 90-degree knee angle, with the supporting leg performing a squat to a 60-degree knee flexion angle.
- Single Leg Squat – Back: Hands placed on the hips, the non-supporting leg extended backward and bent at a 90-degree knee angle, with the supporting leg performing a squat to a 60-degree knee flexion angle.

During the execution of the “Single Leg Squat” and its variations, participants ensured continuous foot contact with the ground. Valid trials were considered those in which participants maintained balance throughout all 3 repetitions. If an incorrect execution occurred, participants were asked to repeat the attempt. A 3-minute rest period was observed between trials to prevent muscle fatigue.

**Figure 1.**
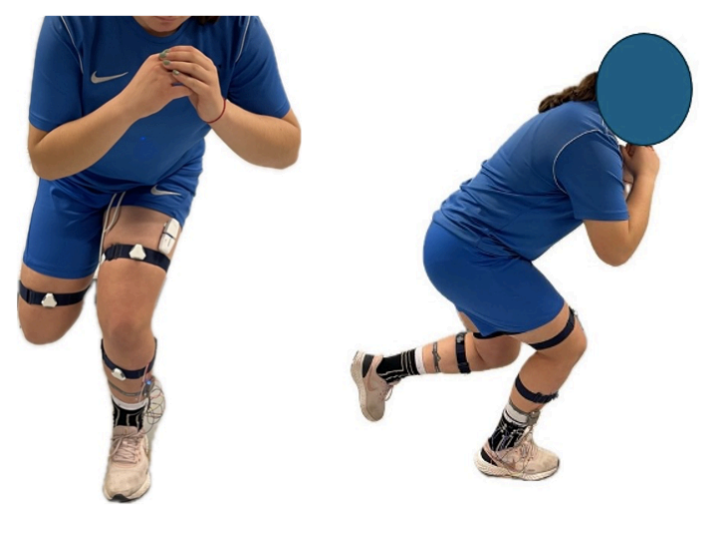
Study participants interacting with the DAid smart sock system, the PLUX Wireless Biosignals (muscleBAN kit) wireless electromyography device, and the NOTCH inertial sensor system.

Throughout the execution of all “Single Leg Squat” tests and their variations, participants wore the DAid smart sock system, the PLUX Wireless Biosignals (muscleBAN kit) for electromyography, and the NOTCH inertial sensor system, as shown in Figure 1.

### Biomechanical Variables for Determining the Movement of the Lower Extremities

Each youth league football player provided data to assess biomechanical movement variables during the “Single Leg Squat” functional test and its variations.

Angles from the NOTCH inertial sensor system: The dataset for the “Lower body+ hip” configuration included dynamic knee valgus determining angles: (1) Angles_Thigh: Flexion and extension, abduction and adduction, and internal and external rotation angles of the hip joint. (2) Angles_LowerLeg: Flexion and extension angles of the knee joint.

COP from the DAid smart sock system: This dataset included COP1X, COP1Y, COP1W, COP2X, COP2Y, and COP2W values, which represent the center of pressure on the plantar surface of the foot. Positive COPX values indicate the medial position, negative values indicate the lateral position, positive COPY values indicate the anterior position, and negative values indicate the posterior position. COPW values represent the total center of pressure. Two mathematical methods (COP1 and COP2) were used to calculate the center of pressure.

Electromyography data from the wireless PLUX system: Electrical activity of VL, GM, GMx*,* and BF and MVC values.

### Data Statistical Analysis

To synchronize datasets from the NOTCH, DAid, and PLUX for biomechanical movement analysis of the lower extremities, data consolidation was performed using the LabView environment. Each participant provided 64 data files, resulting in 2048 files from 32 participants, which were compiled into a unified Database.txt file for correlation analysis. Data analysis was conducted with Microsoft Excel v.16.77.1 and jamovi v.2.3.28.0 (an open-source graphical user interface for the R programming language) using descriptive statistics to describe participant characteristics and results.

Spearman’s correlation analysis, a nonparametric method, was used to determine correlations, considering the Spearman correlation coefficient (ρ). Positive correlations indicate that as one variable increases, so does the other, while negative correlations mean that as one increases, the other decreases. The strength of these relationships is measured by the (ρ up to 0.2 indicates a weak correlation, 0.2 to 0.5 indicates a moderate correlation, and 0.5 to 1 indicates a strong correlation. These correlation strength levels are based on Cohen (1988) [44,45]. Statistical significance was set at *P*<.05, with analyses conducted using jamovi v.2.3.28.0.

## Results

### Participant Description

A total of 32 participants, including 16 female and 16 male individuals, all of whom were youth league football players, were involved in this study. Participants were youth football league athletes who met specific inclusion and exclusion criteria (Table 1).

The positions of the youth league football players (study participants) on the field varied—12 players were defenders, 9 were midfielders, and 11 were forwards.

**Table 1.** Demographic and anthropometric characteristics of youth football players participating in the study (n=32; 16 male and 16 female individuals). Variables include age, body mass index (BMI*)*, height, weight, and European shoe size. These characteristics support contextual interpretation of the biomechanical and sensor data collected during Single Leg Squat testing.

Variable	Mean (SD)	Median	Minimum	Maximum	Unit
BMI	20.8 (2.065)	20.8	17.3	27.2	kg/m²
Age	14.6 (0.495)	15.0	14	15	years
Weight	59.8 (6.413)	58.0	50.0	78.6	kg
Height	169.4 (4.662)	169.0	159	181	cm
EU^a^ Size	40.6 (1.961)	40.0	37.0	44.0	EU shoe size

a EU size: European shoe size measurement.

### Correlation Analysis

#### Strong Positive Correlation

**Figure 2.**
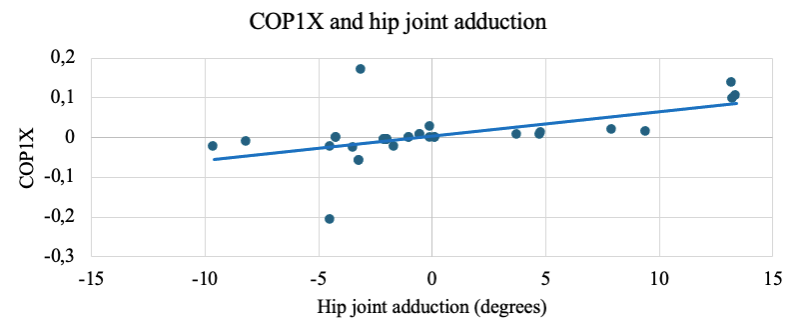
Strong positive correlation (ρ=0.785, *P*<.001) between hip joint adduction and medial plantar pressure (COP1X) during the SLS in youth football players (n=32, aged 14‐15 years). Joint kinematic data were recorded using the NOTCH inertial motion capture system, and plantar pressure was measured using the DAid smart sock system.

A statistically significant relationship was found among the study participants between hip joint adduction movement and changes in the center of pressure on the plantar surface of the foot COP1X (ρ=0.785; *P*<.001), representing the position of the pressure center in the medial part of the plantar surface of the foot (Figure 2).

**Figure 3.**
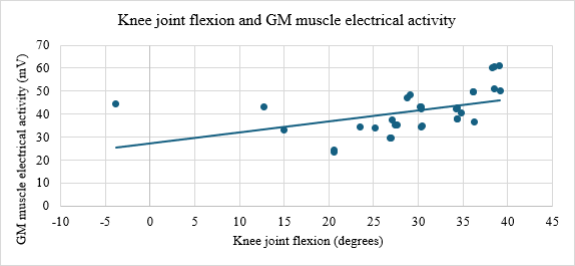
Strong positive correlation (ρ=0.66, *P*<.001) between knee joint flexion and GM muscle activity during the Single Leg Squat test in youth football players. Knee flexion was measured using the NOTCH® inertial sensor system, and muscle activity was recorded via the PLUX Wireless Biosignals surface electromyography system. GM: gluteus medius.

**Figure 4.**
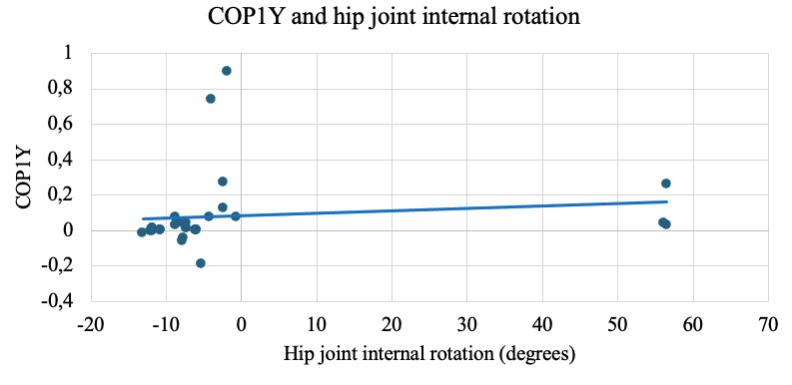
Strong positive correlation (ρ=0.585, *P*<.01) between hip internal rotation and anterior plantar pressure (COPY1) during the SLS. Hip rotation was captured using NOTCH motion sensors, and plantar pressure shifts were measured with the DAid smart sock system.

**Figure 5.**
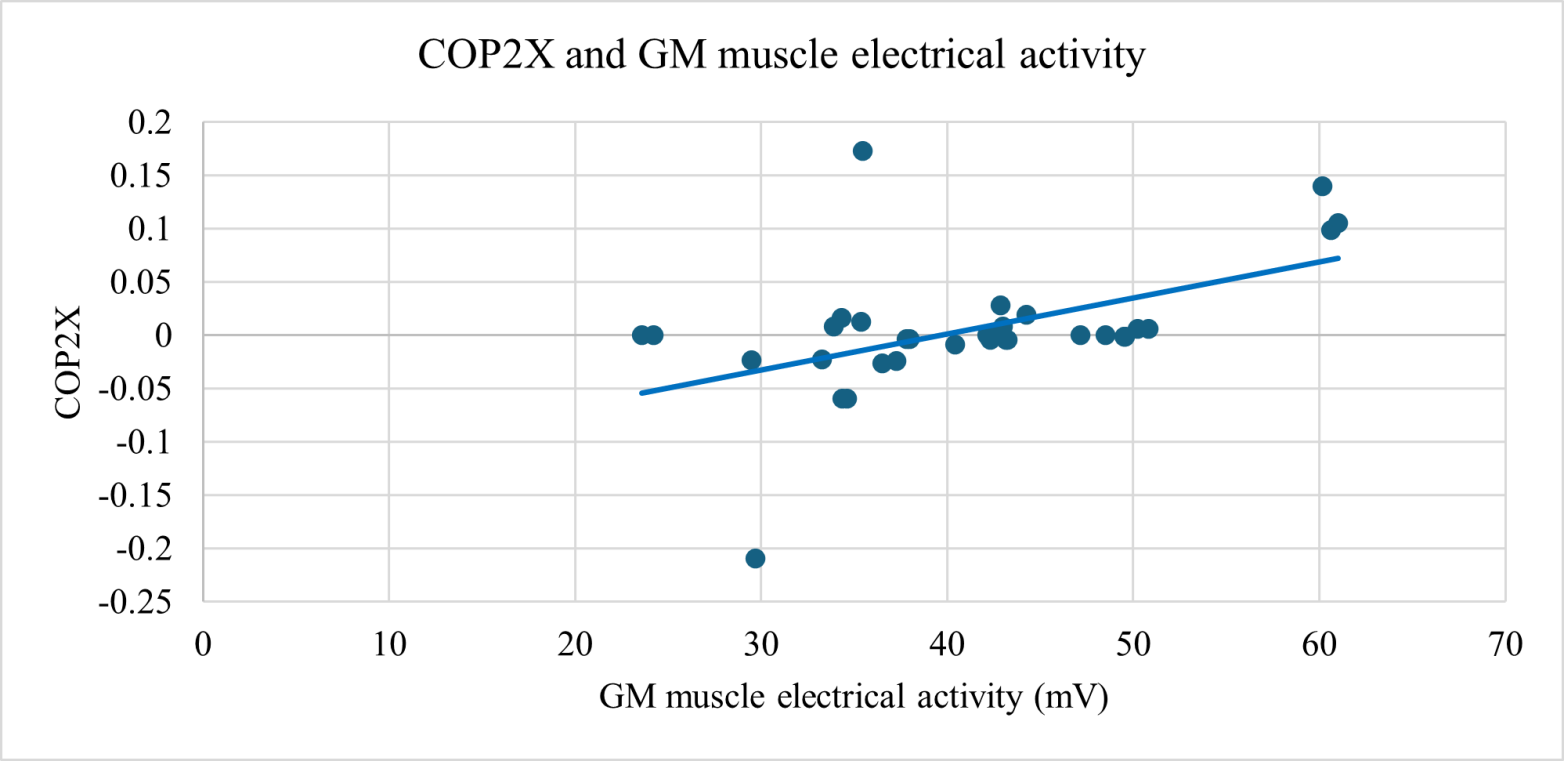
Strong positive correlation (ρ=0.568, *P*<.001) between changes in the center of pressure on the plantar surface of the foot (COP2X) and gluteus medius muscle activity during functional movement assessment. Data were collected using DAid smart socks for pressure and PLUX surface electromyography sensors for muscle activity. GM: gluteus medius.

**Figure 6.**
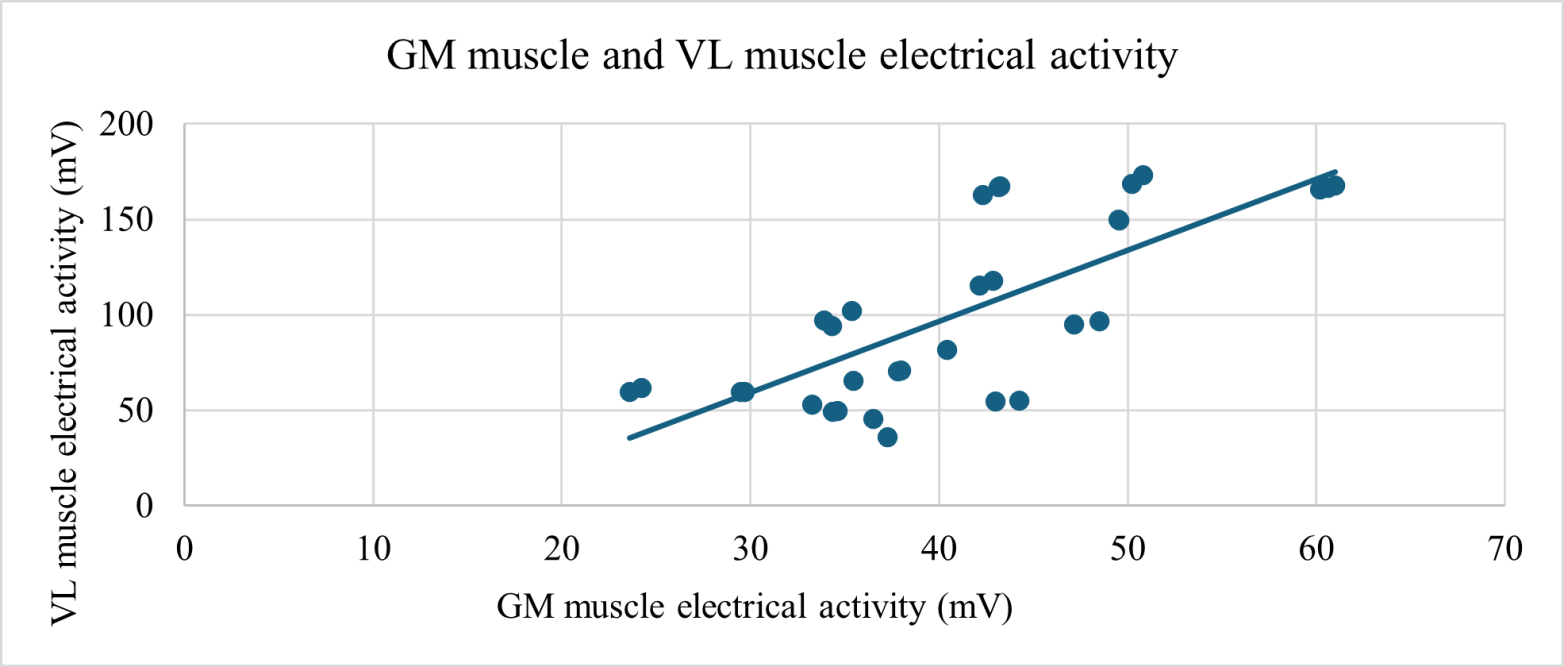
Strong positive correlation (ρ=0.696, *P*<.001) between GM and VL muscle activity during the SLS test. Both muscle signals were recorded using PLUX surface electromyography. GM: gluteus medius; VL: vastus lateralis.

A statistically significant relationship was also found between knee joint flexion and the electrical activity of GM muscle (ρ=0.66; *P*<.001; Figure 3) and between hip joint internal rotation with changes in the COP on the plantar surface of the foot COPY1, representing the position of the pressure center in the anterior part of the plantar surface of the foot (ρ=0.585; *P*<.01; Figure 4), and between changes in the center of pressure on the plantar surface of the foot COP2X, representing the position of the pressure center in the medial part of the plantar surface of the foot, and the electrical activity of GM muscle (ρ=0.568; *P*<.001; Figure 5), and between the electrical activities of the GM and VL muscles (ρ=0.696; *P*<.001; Figure 6).

#### Strong Negative Correlation

**Figure 7.**
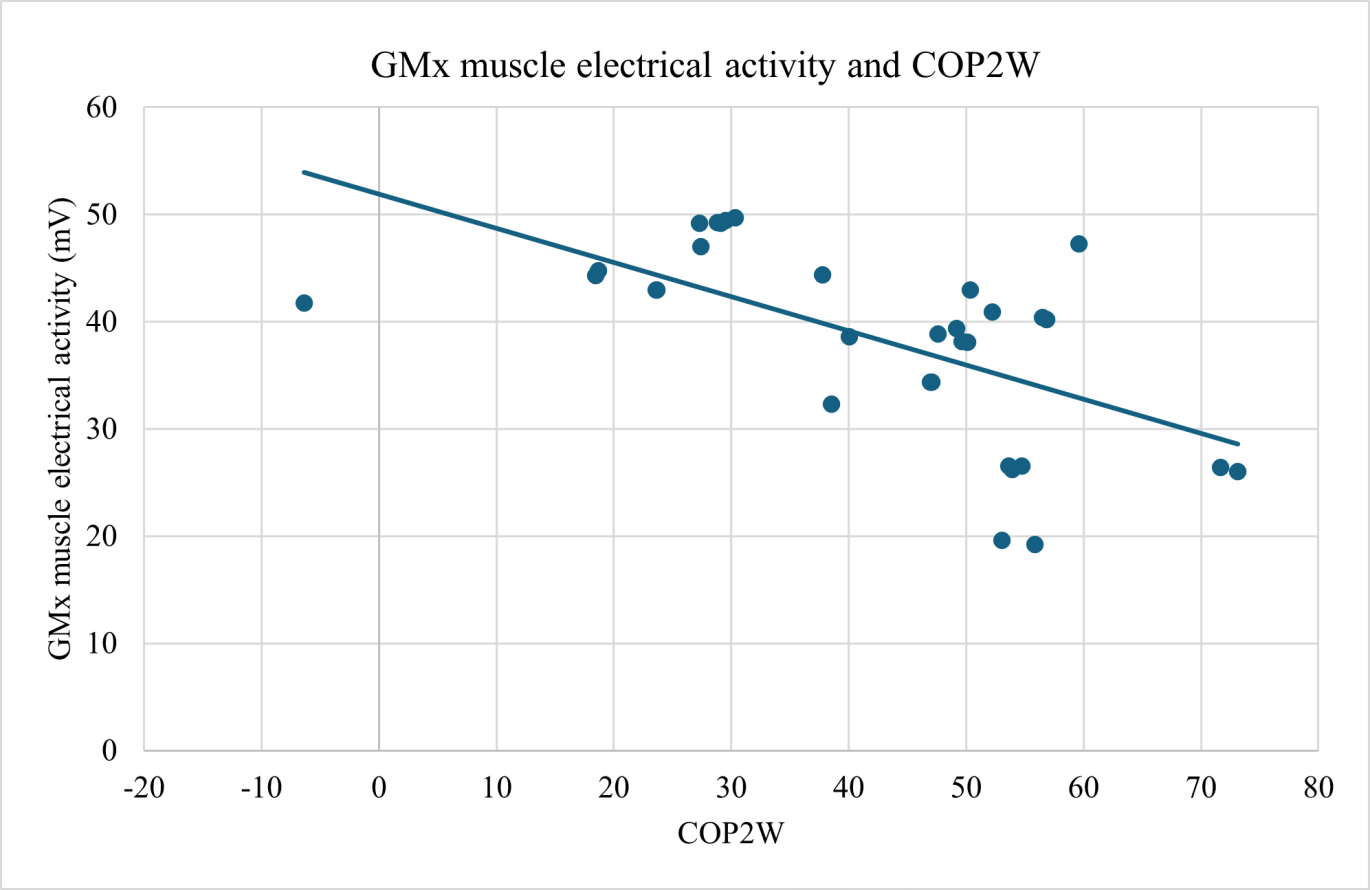
Strong negative correlation (ρ=-0.592, *P*<.001) between overall plantar pressure (COP2W) and GMx muscle activity. Plantar pressure was measured using the DAid smart sock system; muscle activity was measured with PLUX surface electromyography. GMx: gluteus maximus.

**Figure 8.**
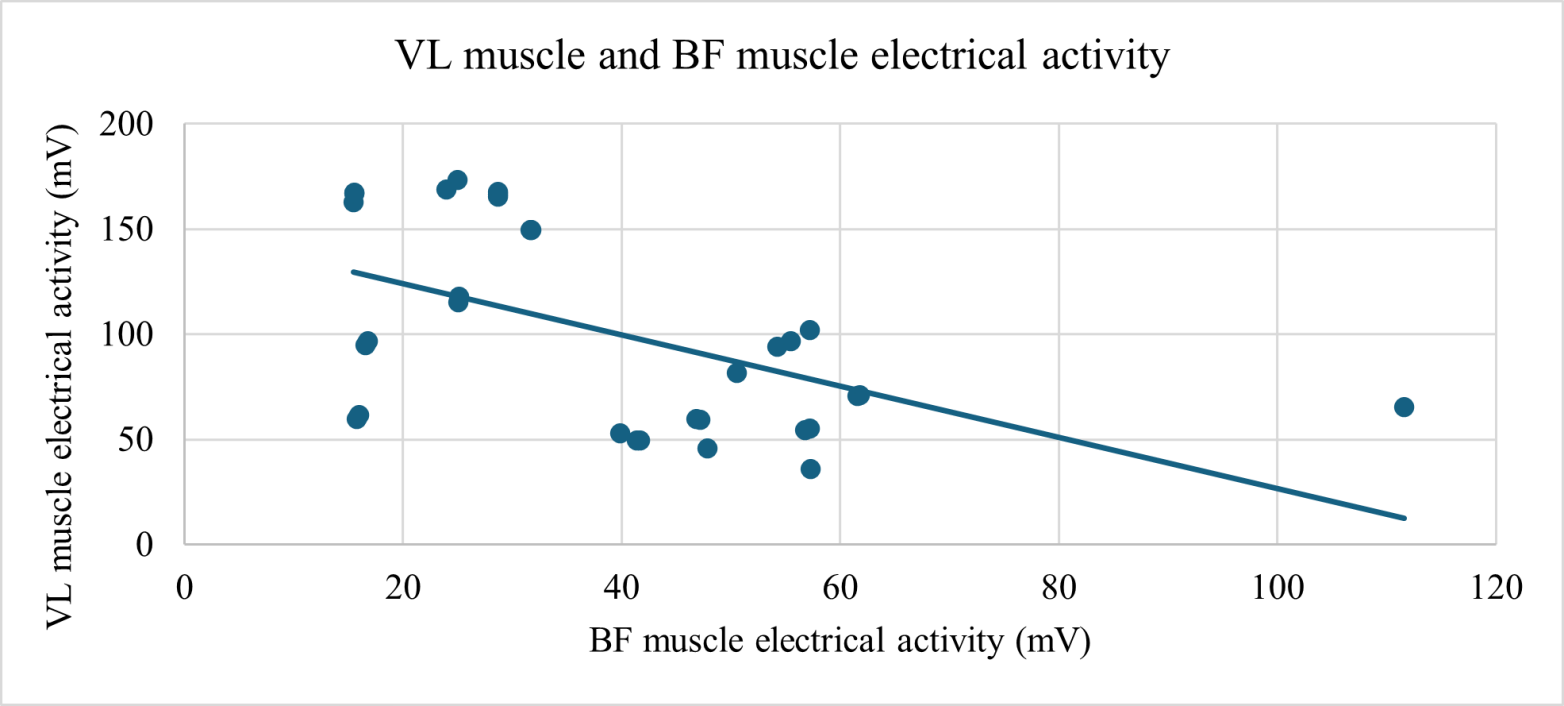
Strong negative correlation (ρ=-0.539, *P*<.01) between BF and VL muscle activation during the SLS. Both muscle activities were assessed using the PLUX Wireless Biosignals surface electromyography system. BF: biceps femoris; GM: gluteus medius; VL: vastus lateralis.

A statistically significant relationship was found among the study participants between changes in the COP on the plantar surface of the foot COP2W, representing the overall position of the pressure center on the plantar surface of the foot, and the electrical activity of GMx muscle (ρ=−0.592; *P*<.001) (see Figure 7) and between the electrical activities of BF and VL muscles (ρ=−0.539; *P*<.01; Figure 8).

#### Moderate Positive and Negative Correlation

Several statistically significant moderate correlations were found among the study participants (Table 2), indicating biomechanical relationships of varying strengths. A moderate positive correlation was observed between hip joint adduction and the electrical activity of the VL muscle (ρ=0.356, *P*<.05), suggesting that increased adduction movement corresponded with greater activation of the vastus lateralis. Similarly, hip joint internal rotation exhibited a moderate positive correlation with the electrical activity of the BF muscle (ρ=0.36, *P*<.05), indicating a link between internal rotation and posterior thigh muscle activation. Changes in the center of pressure on the plantar surface of the foot (COP1Y) were moderately correlated with the electrical activity of the GMx muscle (ρ=0.367, *P*<.05), while the mediolateral shifts of the center of pressure (COP1X) were moderately correlated with anterior-posterior changes in COP1Y (ρ=0.398, *P*<.05), reinforcing the interaction between foot pressure distribution and lower limb movement. Further significant moderate positive correlations were found between COP2X and the electrical activity of the VL muscle (ρ=0.415, *P*<.05), hip joint flexion and COPY2 (ρ=0.426, *P*<.05), and COP2Y and the electrical activity of the BF muscle (ρ=0.426, *P*<.05). The COP1X variable was also moderately correlated with the electrical activity of the GM muscle (ρ=0.445, *P*<.05) and VL muscle (ρ=0.47, *P*<.01), demonstrating a relationship between pressure shifts and muscle activation. Additionally, hip joint internal rotation showed a moderate correlation with the electrical activity of the GMx muscle (ρ=0.477, *P*<.01).

**Table 2.** Moderate positive and moderate negative correlations between joint kinematics, muscle activity, and plantar pressure distribution during the SLS in youth football players. Data were derived using the NOTCH inertial sensor system, DAid smart sock system, and PLUX Wireless Biosignals surface electromyographic. All correlations are statistically significant (*P*<.05 or *P*<.01*).*

Correlation type	First parameter	Second parameter	Coefficient (ρ)	Significance (*P* values)
Moderate positive				
	Hip joint adduction (°)^a^	VL^b^ muscle electrical activity (mV)^c^	0.356	<.05
Hip joint internal rotation (°)	BF^d^ muscle electrical activity (mV)	0.36	<.05
COP1Y (a.u.)^e^	GMx^f^ muscle electrical activity (mV)	0.367	<.05
COP1X (a.u.)	COP1Y (a.u.)	0.398	<.05
COP2X (a.u.)	VL muscle electrical activity (mV)	*r*=0.415	<.05
Hip joint flexion (°)	COPY2 (a.u.)	0.426	<.05
COP2Y (a.u.)	BF muscle electrical activity (mV)	0.426	<.05
COP1X (a.u.)	GM^g^ muscle electrical activity (mV)	0.445	<.05
COP1X (a.u.)	VL muscle electrical activity (mV)	0.47	<.01
Hip joint internal rotation (°)	GMx muscle electrical activity (mV)	0.477	<.01
Moderate negative				
	Hip joint internal rotation (°)	COP2W (a.u.)	−0.368	<.05
COP1W (a.u.)	Hip joint internal rotation (°)	-0.39	<.05
COP2Y (a.u.)	GMx muscle electrical activity (mV)	−0.394	<.05
COP2X (a.u.)	COP2W (a.u.)	−0.415	<.05
COP1Y (a.u.)	COP2W (a.u.)	−0.437	<.05

a (°): degrees - Joint angles.

b VL: vastus lateralis.

c (mV): millivolts.

d BF: biceps femoris.

e (a.u.): arbitrary units.

f GMx: gluteus maximus.

g GM: gluteus medius.

Moderate negative correlations were also identified in the study. Hip joint internal rotation was negatively correlated with COP2W (ρ=-0.368, *P*<.05), indicating that increased internal rotation was associated with a posterior shift in the pressure center. A negative correlation was also observed between the overall pressure center COP1W and hip joint internal rotation (ρ=-0.39, *P*<.05). In addition, COP2Y exhibited a moderate negative correlation with the electrical activity of the GMx muscle (ρ=-0.394, *P*<.05), while COP2X and COP2W were inversely related (ρ=-0.415, *P*<.05). Another significant moderate negative correlation was found between COP1Y and COP2W (ρ=-0.437, *P*<.05), indicating an interaction between anterior-posterior shifts in plantar pressure distribution.

#### Correlation Matrix Analysis by Gender

The study also examined correlations based on gender distribution (see Table 3).

**Table 3.** Correlation matrix for male youth football players (n=16), showing strong positive and negative associations between hip and knee joint angles, plantar pressure distribution, and muscle activity during the Single Leg Squat test. Measures were collected using a multi-sensor setup: NOTCH® for motion, DAid® for pressure, and PLUX for surface electromyography.

Correlation type	First parameter	Second parameter	Coefficient (ρ)	Significance (*P* values)
Strong positive				
	Hip joint adduction (°)^a^	Hip joint internal rotation (°)	0.806	<.001
	Hip joint adduction (°)	COP1X (a.u.)^b^	0.785	<.001
	Knee joint flexion (°)	GM^c^ muscle electrical activity (mV)	0.809	<.001
	Knee joint flexion (°)	GMx^d^ muscle electrical activity (mV)	0.841	<.001
	Knee joint flexion (°)	VL^e^ muscle electrical activity (mV)	0.776	<.001
	COP1X (a.u.)	COP2X (a.u.)	0.832	<.001
	COP2X (a.u.)	GM muscle electrical activity (mV)	0.791	<.001
Strong negative	COP1Y (a.u.)	COP1W (a.u.)	-0.832	<.001

a (°): degrees - Joint angles.

b (a.u.): arbitrary units.

c GM: gluteus medius.

d GMx: gluteus maximus.

e VL: vastus lateralis.

Among male participants, a strong positive correlation was observed between hip joint adduction and hip joint internal rotation (ρ=0.806, *P*<.001), indicating that increased adduction movement was associated with greater internal rotation of the hip joint. In addition, hip joint adduction showed a strong positive correlation with changes in the COP on the plantar surface of the foot (COP1X) (ρ=0.785, *P*<.001), suggesting that medial shifts in the pressure center were linked to increased hip adduction. Knee joint flexion demonstrated a strong positive relationship with the electrical activity of the GM muscle (ρ=0.809, *P*<.001), GMx muscle (ρ=0.841, *P*<.001), and VL muscle (ρ=0.776, *P*<.001), confirming that increased knee flexion corresponded with heightened muscle activation in these regions. Moreover, a strong correlation was found between the changes in the COP variables COP1X and COP2X (ρ=0.832, *P*<.001), indicating a strong interaction between these mediolateral pressure shifts.

Similarly, COP2X showed a strong positive correlation with the electrical activity of the GM muscle (ρ=0.791, *P*<.001), reinforcing the link between plantar pressure adjustments and muscle activation patterns. A strong negative correlation was found between COP1Y and COP1W (ρ=-0.832, *P*<.001), suggesting that anterior-posterior shifts in the COP were inversely related to overall pressure distribution.

For female participants, a strong positive correlation was identified between knee joint flexion and the overall center of pressure on the plantar surface of the foot (COP2W; ρ=0.818, *P*<.001).

## Discussion

### Principal Results

The key findings of this study reveal strong positive and negative correlations between lower limb biomechanics during the “Single Leg Squat” in youth football players, using wireless sensor systems. These results provide important biomechanical insights that may help in identifying movement patterns associated with increased injury risk. Understanding the interaction between foot pressure distribution, joint angles, and muscle activity can guide targeted interventions to improve stability and prevent common football-related injuries, such as ACL injuries and patellofemoral pain syndrome [46,47]. A study by Ahn et al [21] demonstrated that altered foot biomechanics, particularly excessive pronation, may lead to compensatory changes in hip mechanics, such as increased internal hip rotation and hip adduction. This suggests that foot pronation may lead to altered load distribution and neuromuscular adaptations at the hip joint. As a result, foot mechanics play a crucial role in shaping lower limb kinematics and should be carefully considered when designing training programs for young athletes [21,48]. Similarly, Kim et al [10] found that foot pronation is associated with dynamic knee valgus, which involves a combination of hip adduction and internal rotation, contributing to medial knee displacement and increased risk of lower limb injuries. The findings of this study align with those of Kim et al [10], demonstrating that a medial shift in the COP was associated with hip adduction and internal rotation, supporting the link between altered foot mechanics and knee valgus in dynamic movements. Our study further supports these associations, reinforcing the idea that foot pressure analysis can be a valuable screening tool for injury risk assessment. Findings from the DAid smart sock system reinforced the link between medial plantar pressure distribution and hip adduction, which aligns with studies by Sanchis et al [49] and Tran et al [50]. Similarly, hip internal rotation was associated with increased muscle activity in the gluteus maximus and biceps femoris, supporting the role of proximal muscle engagement in dynamic lower limb stability [51]. These findings emphasize the importance of strengthening the hip and hamstring muscles in football players, as improved proximal control may help mitigate excessive knee valgus and reduce injury risk [52]. Further examination of muscle activity patterns reveals the role of neuromuscular control in maintaining lower limb stability. For instance, gluteus maximus and biceps femoris activation correlated with hip internal rotation, which is consistent with previous findings by Wilczynski et al [51] suggesting that proximal muscle control influences knee joint alignment during functional tasks [53]. Increased dynamic knee valgus was associated with higher gluteus maximus activity, reinforcing earlier studies on the importance of hip muscle engagement in knee biomechanics and valgus control [54,55]. This aligns with findings from a systematic review, which highlighted the association between gluteus maximus activation and hip and knee control during single-leg tasks [56], as well as research indicating that higher gluteal activation levels are linked to improved functional performance in tasks requiring lower limb stability and power, such as the 6-meter timed hop test [57]. From a practical perspective, these results indicate that strength and neuromuscular training focusing on the gluteus maximus and hamstrings could be beneficial for reducing valgus-related injuries in youth athletes [58]. While the current study focused on lower limb kinematics and pressure distribution, future research should further explore the dynamic interactions between foot posture, knee mechanics, and proximal muscle function in movement stability. Incorporating real-time feedback using wireless sensor systems during training may enhance movement efficiency and reduce injury risk, an area that warrants further investigation [59,60].

### Limitations

This study highlights key limitations that should be addressed in future research. One important aspect is the distinction between muscle activation and muscle force generation. While surface electromyography provides valuable insights into muscle activity, it does not directly quantify neural drive or muscle force [61]. The relationship between these variables is influenced by multiple factors, including electrode positioning, motor unit recruitment, and tissue conductivity, making it difficult to infer strength solely from electromyographic amplitude. Future studies should further investigate neuromuscular control strategies and their implications for lower limb biomechanics, ensuring that clinical applications account for these complexities and uncertainties [61,62]. In addition, this study focused on youth football players aged 14‐15 years old, a group at higher risk of neuromuscular adaptations and injury [4]. While these findings provide valuable insights into adolescent biomechanics, broader age groups and different sports, such as basketball, should be included in future research to determine the generalizability of the results. Future research could include individuals with lower limb pathologies, such as patellofemoral pain syndrome or postanterior cruciate ligament reconstruction, to provide data on biomechanical parameters and their relationships during functional tasks, aiming to identify risk factors and improve rehabilitation strategies to reduce the risk of progressive lower limb injuries. The research design and methodology effectively captured key biomechanical associations; however, additional experimental conditions—such as fatigue testing or perturbation trials—could provide deeper insights into neuromuscular responses under dynamic conditions.

### Comparison With Previous Work

The use of wireless sensor systems in this study allowed for detailed in-field analysis of lower limb biomechanics, but alternative technologies could further refine measurement accuracy and expand analytical capabilities. For instance, the PLUX Wireless Biosignals (muscleBAN kit) was used to assess muscle activation, but the Ultium Wireless Surface Electromyographic system could offer even greater resolution, with its high sampling rates, low noise levels, and SmartLead options, making it a strong alternative for future research [63]. The ability to capture real-time movement data outside laboratory settings provides a significant advantage for applied sports science [19]. These results demonstrate that wearable sensors can provide meaningful biomechanical insights without the constraints of traditional motion capture systems, making them more feasible for training environments [64]. Similarly, the NOTCH inertial sensor system effectively captured lower limb kinematics, but alternative motion capture systems such as OptiTrack could provide more precise 3D movement analysis [65,66]. However, considering the practical challenges of optical motion capture in sports settings [65,66], the use of wearable inertial sensors presents a valuable alternative for continuous monitoring and individualized movement assessment [67].

### Gender-Based Biomechanical Differences

Among male participants, hip adduction and hip internal rotation showed a strong correlation, which suggests that males may be more reliant on hip-dominant movement strategies for single-leg stability [56]. This could have implications for training programs, as focusing on hip abductor and external rotator strength may help correct excessive hip adduction patterns that could lead to knee valgus [51,68]. For female participants, the most significant correlation was between knee joint flexion and the overall COP (COP2W). This suggests that female athletes may demonstrate different weight distribution strategies during squatting movements, potentially increasing their vulnerability to ACL injuries [57]. Given that previous research has highlighted greater valgus angles in female athletes, our findings suggest that targeted neuromuscular training, including balance and landing mechanics, may help mitigate these risks [69]. In addition, studies have demonstrated that eccentric hip abductor and lateral rotator strength are significantly associated with lower limb kinematics, particularly in women, reinforcing the importance of proximal control in mitigating excessive knee valgus and femur adduction [69]. Future research should further investigate these gender-based variations in lower limb biomechanics to better understand potential injury risk factors and inform targeted training interventions for male and female athletes [70]. Coaches and physiotherapists could use this information to create individualized training plans to optimize performance while minimizing injury risk.

### Conclusions

This study identified significant correlations between lower limb biomechanics during the “Single Leg Squat” functional test in youth football players. Strong associations were observed between foot COP, lower limb movement patterns, and muscle activity, demonstrating the feasibility of using wireless sensor systems for biomechanical assessment in field conditions. The findings confirm that wireless sensor technology, including the DAid smart sock system, provides reliable data for evaluating lower limb biomechanics in football players.

## Abbreviations

ACL: anterior cruciate ligament
BF: biceps femoris
COP: center of pressure
FIFA: Fédération Internationale de Football Association
GM: gluteus medius
GMx: gluteus maximus
MVC: maximum voluntary contraction
SENIAM: Surface Electromyography for the Non-Invasive Assessment of Muscles
SLS: Single Leg Squat
VL: vastus lateralis
